# O_2_-sensitive microcavity arrays: A new platform for oxygen measurements in 3D cell cultures

**DOI:** 10.3389/fbioe.2023.1111316

**Published:** 2023-02-20

**Authors:** Christoph Grün, Jana Pfeifer, Gregor Liebsch, Eric Gottwald

**Affiliations:** ^1^ Institute of Functional Interfaces, Karlsruhe Institute of Technology, Karlsruhe, Germany; ^2^ PreSens Precision Sensing GmbH, Regensburg, Germany

**Keywords:** oxygen measurement, spheroid, organoid, fluorescence quenching, mito stress test, 3D cell culture, microcavity array

## Abstract

Oxygen concentration plays a crucial role in (3D) cell culture. However, the oxygen content *in vitro* is usually not comparable to the *in vivo* situation, which is partly due to the fact that most experiments are performed under ambient atmosphere supplemented with 5% CO_2_, which can lead to hyperoxia. Cultivation under physiological conditions is necessary, but also fails to have suitable measurement methods, especially in 3D cell culture. Current oxygen measurement methods rely on global oxygen measurements (dish or well) and can only be performed in 2D cultures. In this paper, we describe a system that allows the determination of oxygen in 3D cell culture, especially in the microenvironment of single spheroids/organoids. For this purpose, microthermoforming was used to generate microcavity arrays from oxygen-sensitive polymer films. In these oxygen-sensitive microcavity arrays (sensor arrays), spheroids cannot only be generated but also cultivated further. In initial experiments we could show that the system is able to perform mitochondrial stress tests in spheroid cultures to characterize mitochondrial respiration in 3D. Thus, with the help of sensor arrays, it is possible to determine oxygen label-free and in real-time in the immediate microenvironment of spheroid cultures for the first time.

## 1 Introduction

Cells have been cultivated in 2D for more than a century (Altmann et al., 2009; Jensen and Teng, 2020) and due to their advantages, such as inexpensiveness and easy handling, they have been the standards in most assays. However, 2D culture approaches suffer from several limitations. In particular, due to differences in cell-cell or cell-matrix contacts, lacking of a proper polarization, as well as hyperoxic culture conditions, 2D cultured cells cannot properly simulate a tissue and therefore, are not able to mimic the *in vivo* situation. This is especially true for nutrient gradients, proliferation, morphology, or even signal transduction (Huh et al., 2011; Lv et al., 2017; Badr-Eldin et al., 2022).

Both, fundamental research and drug development are in an urgent need of cell culture models that reflect physiological conditions more reliably, thus a number of 3D cell culture systems have been established in the last decades. These organotypic models also promise to reduce the number of animal tests in drug development (Shen et al., 2021).

3D cell cultures can be classified into scaffold-free and scaffold-based methods. Scaffold-free 3D cell cultures include (multi) cellular spheroids, which can be generated, e.g., by hanging drop methods, in spinner flasks, or multiwell plates that feature a non-adhesive U- or V-shaped bottom and which have become of widespread use (Buentello et al., 2021). Scaffold-based 3D cell culture models comprise hydrogels, made of extracellular matrix components or synthetic structures, such as polymer scaffolds (Justice et al., 2009; Badr-Eldin et al., 2022). A further step towards a higher complexity comprises those 3D cell cultures that can be cultivated in microbioreactors or organ-on-chip systems since, e.g., an active flow can be applied (Huh et al., 2011; Grün et al., 2020; Cacciamali et al., 2022). At the Karlsruhe Institute of Technology, the so-called microcavity array system has been developed. It is based on a 50 µm thin polymer film with an area of 1 cm^2^ containing several hundred microcavities with a typical diameter of 300 µm and a depth of up to 300 µm in which cells/organoids can be cultivated three-dimensionally. These arrays are generated by a microthermoforming process (Giselbrecht et al., 2006; Gottwald et al., 2007; Giselbrecht et al., 2008). Due to the fixed position and the large number of microcavities, the system is also suitable for high-throughput applications (Nies et al., 2019). As mentioned earlier, one advantage of 3D cell culture models is the fact that oxygen gradients can be formed, along with others, such as those of nutrients, metabolites, and waste molecules. However, the oxygen concentrations occurring in 3D cell cultures do not always reflect the *in vivo* situation. A large majority of experiments to date are performed under ambient atmosphere, which translates into hyperoxic culture conditions, or with extremely hypoxic values rather than with the tissue-typical oxygen concentrations (Al-Ani et al., 2018; Tse et al., 2021). Moreover, typical tissue oxygen concentrations are sometimes not known because they can hardly be determined although they are essential for the maintenance of tissue-specific metabolism (Carreau et al., 2011; Zhang et al., 2016; Cani et al., 2022). The measurement of oxygen gradients and also the regulation of the oxygen supply of the culture system is still a major challenge but will be necessary to mimic *in vivo*-like oxygen concentrations in the cell culture model. There are several methods to analyze the dissolved oxygen concentration. One of them is the Winkler titration, an improvement of the iodometry (Carpenter, 1965). This technique consumes oxygen and leads to the insoluble precipitate Mn(OH)_2_, rendering the Winkler method not suitable for *in situ* measurements in cell culture. Electrochemical dissolved oxygen sensors, such as Clarke-type electrodes, also consume the analyte and are therefore neither suitable for use in cell culture (Wei et al., 2019). In contrast to the above-mentioned methods, optical oxygen sensors are especially suitable for cell culture applications because they do not consume oxygen during measurement, have a high precision, and can be miniaturized, which also enables their integration in lab-on-chip or organ-on-chip devices (Azimzadeh et al., 2021). Their measuring principle, fluorescence- or phosphorescence-based methods, is becoming increasingly popular. They are based on a quenching effect in which a fluorophore, often a platinum, palladium, or ruthenium complex, is first excited by light of a specific wavelength that, in the presence of oxygen, does not emit the energy in the form of light, but rather is transferred radiation-free to oxygen. Thus, quenching allows the oxygen concentration to be measured (Wang and Wolfbeis, 2014; Rivera et al., 2019b). These oxygen-sensitive fluorophores can directly be bound to a hydrogel, a scaffold, or to nanoparticles (Dmitriev and Papkovsky, 2012; Dmitriev et al., 2014; Jenkins et al., 2015; Ashokkumar et al., 2020), which are taken up by the cells or spheroids and can thus directly influence or affect the cells, for example. Alternatively, the fluorophores can be immobilized on a polymer film (Kellner et al., 2002; Babilas et al., 2005) that can then be used for various applications. For example, oxygen can be measured at a specific point or two-dimensionally over an entire area of the cell culture (Westphal et al., 2017; Rivera et al., 2019a; Wolff et al., 2019). One approach to determine oxygen in three dimensions is to move a sensor along the *Z*-axis. Systems using both optical (Eggert et al., 2021) and electrochemical (Kagawa et al., 2015) needle-based sensors have been described for this purpose. Peniche Silva et al. (2020) described a system where they used an oxygen-sensitive film fixed on a ramp in order to measure oxygen gradients and profiles above a monolayer of cells. However, most systems are not really suited for the use in 3D cell culture. For example, measuring oxygen in the microenvironment of spheroids with needle-based sensors, as described by Eggert et al. (2021) or Kagawa et al. (2015), requires an enormous amount of equipment. Also, the parallel investigation of several spheroids is not possible. The system of Peniche Silva et al. (2020) would also allow for the introduction of 3D cell cultures, however the system would not allow measurements in the immediate microenvironment of the cells.

In this work, we introduce a method that combines the microcavity array 3D culturing technique with that of a fluorophore-based oxygen sensing *via* microthermoformed oxygen-sensitive polymer films. This approach allows us to create a 3D cell culture system (sensor arrays) in which we can generate and culture a large number of spheroids in parallel and, for the first time, determine the oxygen level label-free and in real-time in the microenvironment of single spheroids and, in principle, of a complete spheroid array. In pilot experiments, we evaluated different culture conditions using HepG2 cells and performed oxygen measurements during cultivation. In addition, various parameters of mitochondrial functions were measured in a proof-of-concept series of experiments, that currently can only be performed in 2D cell cultures according to the so-called Mito Stress Test (Agilent Technologies). The use of spheroids in the above-mentioned experimental setup is of limited use because oxygen can only be measured globally (well). Currently, our system is able to measure profiles of mitochondrial respiration on a single spheroid/organoid level and determines the basal respiration, the ATP-linked respiration, and the maximal respiration of up to 120 aggregates/organoids at a time and that number can easily be upscaled.

## 2 Methods

### 2.1 Design and manufacturing of oxygen sensor arrays

For the manufacturing of the sensor arrays we used a standard polycarbonate film (it4ip) with a thickness of 50 µm and that was coated with an oxygen-sensitive fluorescent dye incorporating polymer film (fluorescent optical sensor) supplied by PreSens Precision Sensing GmbH. A microthermoforming system, developed at the Karlsruhe Institute of Technology, was used to generate the sensor arrays. For this process, the sensor films were placed on top of a brass molding mask and formed into microcavity arrays by applying 20 bars at a temperature of 152.5°C. The microcavity arrays were further characterized by using a standard bright field microscope that was equipped with a micrometer screw attached to the microscope base, so that the microcavity depths could be determined and which was done at 4 representative positions of each array. The results were further validated with a laser scanning microscope for material characterization (Keyence VHX5000).

### 2.2 Cells and culture conditions

HepG2 cells were cultured under standard conditions in a humidified atmosphere containing 5% CO_2_ and 18.6% O_2_ at 37°C in T75 cell culture flasks. The cells were passaged as needed, but were usually split every 3–4 days at a ratio of 1:3. MEM medium (ThermoFisher Scientific) containing 10% FBS, 1% non-essential amino acids solution (NEAA, ThermoFisher Scientific), 1% GlutaMAX™ (ThermoFisher Scientific), 1%, Penicillin-Streptomycin (ThermoFisher Scientific), and 0.1% phenol red (ThermoFisher Scientific), hereafter referred to as HepG2 medium, was used for the standard culture. HepG2 medium without phenol red was used for the oxygen measurements and is referred to as assay medium.

### 2.3 Cell staining and fluorescence microscopy

Live cell staining was performed with CellTracker™ green (ThermoFisher Scientific). For this, the cells were incubated with 1 µM CellTracker™ green for 30 min before seeding. Afterwards, the cells were washed once with PBS^+/+^ and seeded at a density as needed for the experiment duration. Staining with SYTO™16 (ThermoFisher Scientific) was done immediately before microscopy by incubating the cells/spheroids with 5 µM SYTO™16 for 45 min. This was followed by a medium change. Propidium iodide (Biotium, 1 μM, 45 min) was used for counterstaining dead cells. After staining, the cells were washed and immediately examined under a fluorescence microscope.

A Leica TCS SP5 confocal microscope with an argon laser for 488 nm excitation and a diode laser for 561 nm excitation wavelength was used for microscopy of the different coating types. For z-stacks, the step-size was 5 µm and comprised 80 steps. The hanging drop spheroids were observed on a Zeiss fluorescence microscope with appropriate filter sets.

### 2.4 Assembly of the cell culture inserts and coating

After thermoforming, the sensor array was cut by punching to a diameter of 16 mm. To sterilize the arrays and to remove air bubbles from the microcavities, the array was subjected to an isopropanol series consisting of 100%, 70%, 50%, and 30% isopropanol for 15 s, respectively, and afterwards washed twice with sterile, demineralized water. The arrays prepared in this way were placed into a CellCrown™ 12NX (Scaffdex) cell culture insert and placed in one well of a 12-well cell culture plate. For coating with BIOFLOAT™ (FaCellitate), the arrays were sterilized in 70% isopropanol for 15 s, mounted into the CellCrown™ holder, and then air dried.

Collagen coating was performed by diluting 18 µl of a collagen stock solution [2 mg/ml in 0.2% (v/v) acetic acid] with 132 µl of PBS^−/−^ (ThermoFisher Scientific). 150 μl of this working solution were placed on top of the sensor array and incubated for 1 h under standard incubation conditions. Afterwards, the array was washed twice with PBS^+/+^ (ThermoFisher Scientific).

For coating with BIOFLOAT™ (FaCellitate), 300 µl of the solution were added to the sensor array which was then centrifuged at 300 g for 1 min. The cell culture insert was incubated for 5 min at room temperature. Afterwards, the arrays were air dried under the clean bench for at least 30 min. 2 ml assay medium were added into the cell culture insert as well as into the plate well below. Air bubbles were removed from the cavities by gentle tapping, the use of negative pressure by using an adapter and a vacuum pump, or centrifugation at 300 g for 1 min. In addition, 2 ml of sterile water were added to the surrounding wells to ensure proper humidity during the cultivation.

### 2.5 Generation of spheroids

Prior to the inoculation of the cells, the medium in the cell culture insert as well as in the plate well was removed, except for 200 µl that remained in the well to avoid air bubble formation below the insert.

Spheroids were generated in two different ways. For a direct generation of cell aggregates within the microcavities, 50.000–150.000 HepG2 cells, depending of the duration of the experiment, were added in 40 µl assay medium on top of the dried center of the sensor array in the cell culture insert. Cells were incubated at 37°C, 5% CO_2_ for 15 min in order to sediment the cells into the microcavities. Afterwards, 550 µl of HepG2 or assay medium were added to the insert as well as to the well compartment below.

Alternatively, spheroids were generated by the hanging drop method. For this, assay media drops with a volume of 25 µl containing 625 cells, were pipetted onto the inner lid surface of a 10 cm petri dish. To prevent evaporation, 5 ml PBS^−/−^ were added to the petri dish. The cells in the drops were incubated for 4 days to ensure a proper spheroid generation. After spheroid formation, the drops were collected, the spheroids were allowed to sediment, the supernatant was removed, the spheroids were resuspended in 40 µl medium, and as such, pipetted on top of the sensor arrays. After 5 min for the sedimentation of the spheroids, their positions in the microcavities were checked under the microscope after which additional 550 µl medium were added to each well and insert, respectively.

### 2.6 Sensor film calibration

A two-point calibration was performed to calibrate the sensor array foil. For the maximum value of dissolved oxygen, a drop of 100 µl water was placed on the foil surface, equilibrated for 10 min in the incubator under experimental conditions after which an image was taken with the help of the VisiSens system (PreSens Precision Sensing GmbH). The ratio of the red and the green fluorescence signal of this image corresponds to the maximum soluble oxygen and is subsequently referred to as 100% oxygen. For the zero-point calibration (0% oxygen), one drop (100 µl) of a sodium sulfite solution (10 mg/ml) was pipetted on top of the foil. The ratio (*R*) of the red and green fluorescent signal of the sensor foil was used to create a calibration plot from both points (Klimant et al., 1995; Tschiersch et al., 2012), which can be used to convert the measured values into oxygen concentrations.

### 2.7 Real-time oxygen measurements in 3D cell cultures

To measure the dissolved oxygen concentration during cultivation, the 12-well plates with the inserts were placed on the VisiSens system stage (PreSens), the illumination parameters were set according to the calibration parameters. Images were recorded, depending on the experimental setup, every 1–60 min.

### 2.8 Characterization of mitochondrial respiration

The characterization of the mitochondrial respiration was executed in a humidified atmosphere without additional CO_2_. Spheroids were prepared 3–4 days prior to the experiment. Four modulators of the electron transport chain (ETC) were added sequentially. Images were taken every 2 min. In particular, the spheroids were first incubated for 30 min without any treatment in assay medium including 10 mM HEPES to determine the basal level of respiration. Then, assay medium including 10 mM HEPES was added as a control. After 30 min, 4 µM oligomycin, a complex V inhibitor, was added to determine the ATP-linked respiration. The measurement continued for another 30 min. Afterwards, 2 µM carbonyl cyanide-4 (trifluoromethoxy) phenylhydrazone (FCCP), an uncoupling agent, was added to determine the maximum respiration capacity. After another 30 min, 1 µM of each, rotenone and antimycin A, inhibitors of complex I and III, respectively, were added to inhibit the total respiration of the mitochondria.

### 2.9 Analysis with CellProfiler™

For a quick analysis, the software plug-in MITOS from PreSens was used. For a more detailed analysis we used the open source CellProfiler™ Image Analysis Software (RRID:SCR_007358) (Lamprecht et al., 2007). For this, the microscope image was split into the red and green channel. The green channel represents the signal of the reference fluorophore, which remains constant during the series of measurements. Therefore, this channel was chosen for the identification of the cavities. First, the illumination of the image was optimized (applying a filter, correcting the illumination, adjusting the intensity). Then, the objects (cavities) were detected by CellProfiler™. Based on the cavities, the regions of interest (ROIs) were defined for the spheroids, the cavity walls, and the chamfer. These were used as masks to extract the intensities of the individual fluorophores in the respective ROIs from the raw data (Figure 3B). Further analysis was done using Microsoft Excel.

## 3 Results

### 3.1 Manufacturing of oxygen sensor arrays

**FIGURE 1:**
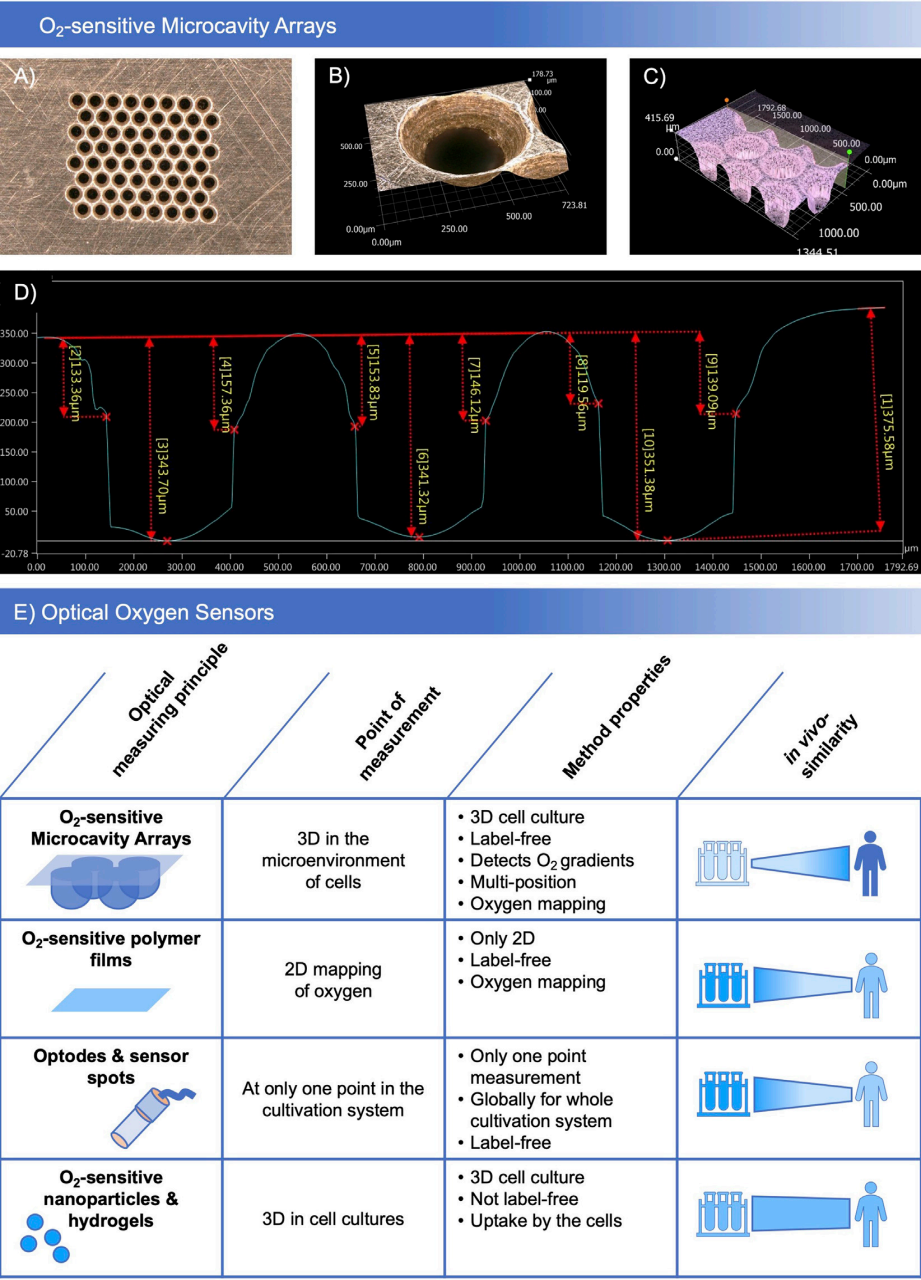
Manufacturing sensor arrays: **(A)** Brass molding mask with 8 × 8 microcavities and a bevel at the upper side of the mask. **(B)** Detailed view of the bevel. **(C)** Microscopic image of the depth profile of the microcavities. **(D)** Characterization of the forming depth. Microscopic images taken with a Keyence VHX 5000 digital microscope, magnification ×20 **(A)** and ×200 **(B,C)**. **(E)** Comparison of O_2_-sensitive microcavity arrays with other established optical oxygen sensors.

In preliminary tests (data not shown), different oxygen sensitive coatings and designs of the molding masks were tested with regards to their microthermoforming process suitability. Polycarbonate-based oxygen-sensitive polymer films showed the best characteristics concerning forming parameters and sensor stability. The different film prototypes showed good biocompatibility and low toxicity for HepG2 and Hela cell lines in preliminary toxicity tests. In sensor material development, different polymer, dye and base film combinations were assigned base film identifiers and an ascending number. Of the tested combinations, SF-RPC3, a sensor foil based on sensor material combination number 3 on polycarbonate base film was identified as the lead material which was selected for further experiments. Depending on the biological application, plasma and non-plasma treated variants of the SF-RPC3 film were used. Figure 1 shows the details of the brass molding mask (Figure 1A) for 8 × 8 microcavity arrays with an inner microcavity diameter of 300 µm. At the upper side, a bevel around each microcavity with an outer diameter of 500 µm was integrated (Figure 1B) which enables the measurement of oxygen gradients. For characterization of the thermoformed structures and optimization of the forming parameters, the profiles of the microcavities were measured (Figures 1C, D). Microthermoforming can be used to produce sensor arrays in a form-accurate, reproducible manner.

### 3.2 Coating method comparison

**FIGURE 2:**
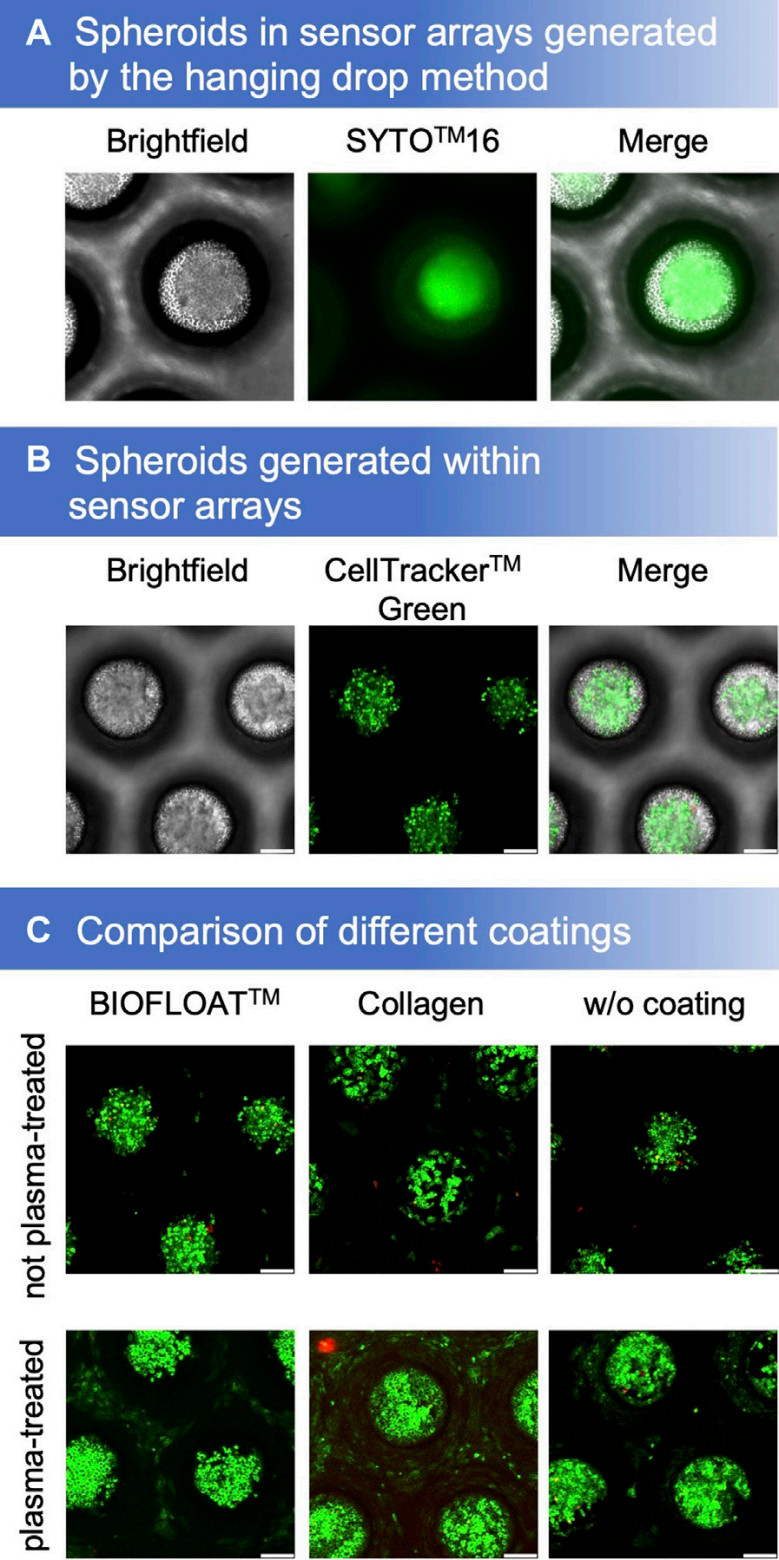
**(A)** Spheroids, stained with Syto™16 and propidium iodide, generated by the hanging drop method could be cultivated in sensor microcavity arrays. **(B)** Another method is to generate spheroids directly in the sensor microcavity array (stained with CellTracker™ green and propidium iodide after 4 days of cultivation). **(C)** Proliferation and aggregation comparison of different SF-RPC3 sensor film coating methods.

By using the hanging drop method, spheroids were obtained that could be cultivated further and analyzed in our microcavity sensor array (Figure 2A). However, it is also possible to generate spheroids directly in the sensor array by inoculating a single-cell suspension (Figure 2B). For cultivation of cells within the oxygen-sensitive microcavities, different coatings on both, argon-plasma treated as well as the non-plasma treated sensor films, were tested. Plasma treatment had a tremendous effect on cell growth. While cells could be cultivated on plasma-treated SF-RPC3 films even without further coating, this was not possible on the non-treated films. Collagen coating is widely used to enable adhesion of cells to polymer surfaces. We could show that cells could be cultured on both the plasma-treated and the non-treated SF-RPC3 films for several days when coated with collagen. The combination of plasma-treatment and collagen-coating was most suitable for adherent cultivation. The influence of the plasma treatment is also clearly visible in the non-coated films. While some cells still grew outside the cavities on the plasma-treated films, this was no more possible on the non-plasma-treated films.

For a convenient 3D cell culture setup, spheroids can be generated directly in the microcavities. However, for this purpose, the cells must not adhere. Therefore, the sensor arrays were coated with BIOFLOAT™ solution. As a result, the cells formed spheroids in the microcavities within several days of culture. Propidium iodide staining after 4 days showed that the surfaces were biocompatible due to the low number of dead cells.

### 3.3 Oxygen measurement in microcavities

**FIGURE 3:**
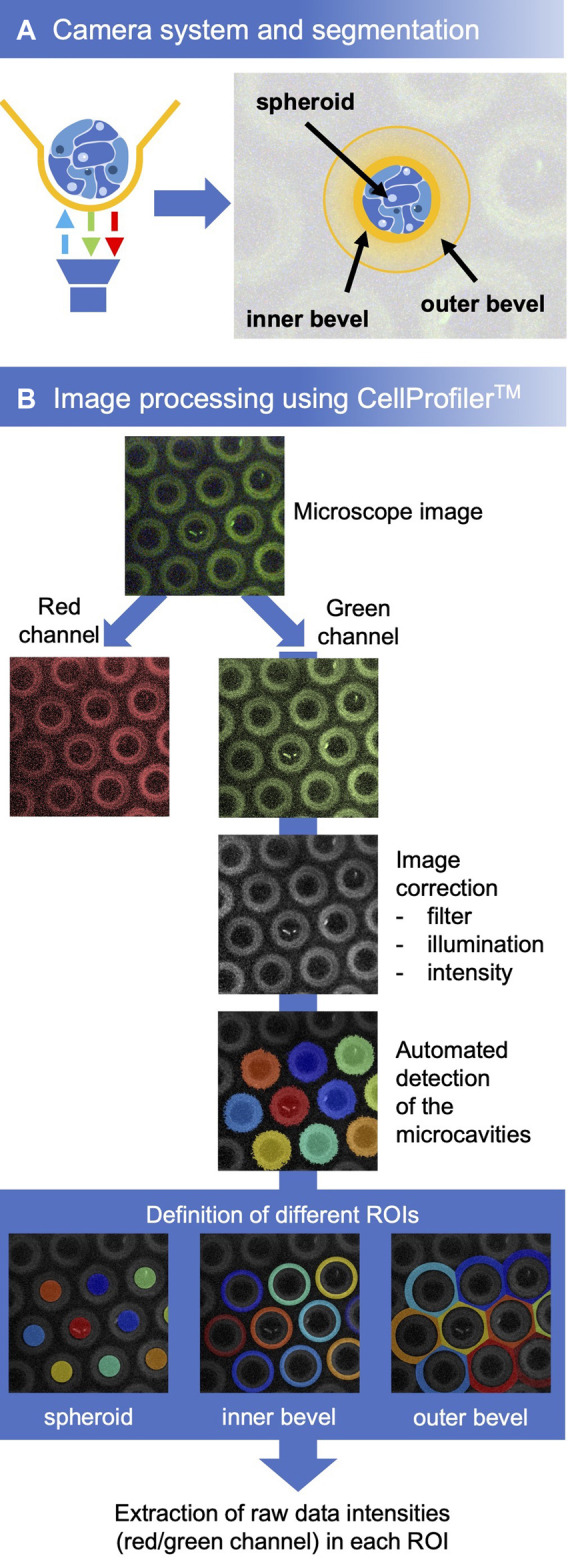
**(A)** Experimental setup and definition of different regions of interest. **(B)** Workflow for the analysis with CellProfiler™. First, the microcavities were detected in the image, subsequently, the three regions of interest were defined. For every ROI of each microcavity, the mean intensity of the red and the green signal was determined after which the oxygen concentrations were calculated with the help of the calibration curve.

Although an analysis of the oxygen concentration was already possible with the help of the plug-ins integrated in the VisiSens software, this method was sensitive to errors because of slight position shifts in the microcavity images that were not automatically corrected during the measurement. Therefore, we developed a protocol, based on the CellProfiler™ software modules, that adapt the image quality, automatically detects objects (the microcavities) and defines the different regions of interest (ROI) based on these objects (Figures 3A, B). To obtain information about the gradients in the microenvironment of the spheroids, the bevel was defined as one ROI. Another one corresponded to the direct microenvironment of the spheroid at the bottom of the microcavity.

### 3.4 Proof-of-concept: Characterization of mitochondrial respiration

**FIGURE 4:**
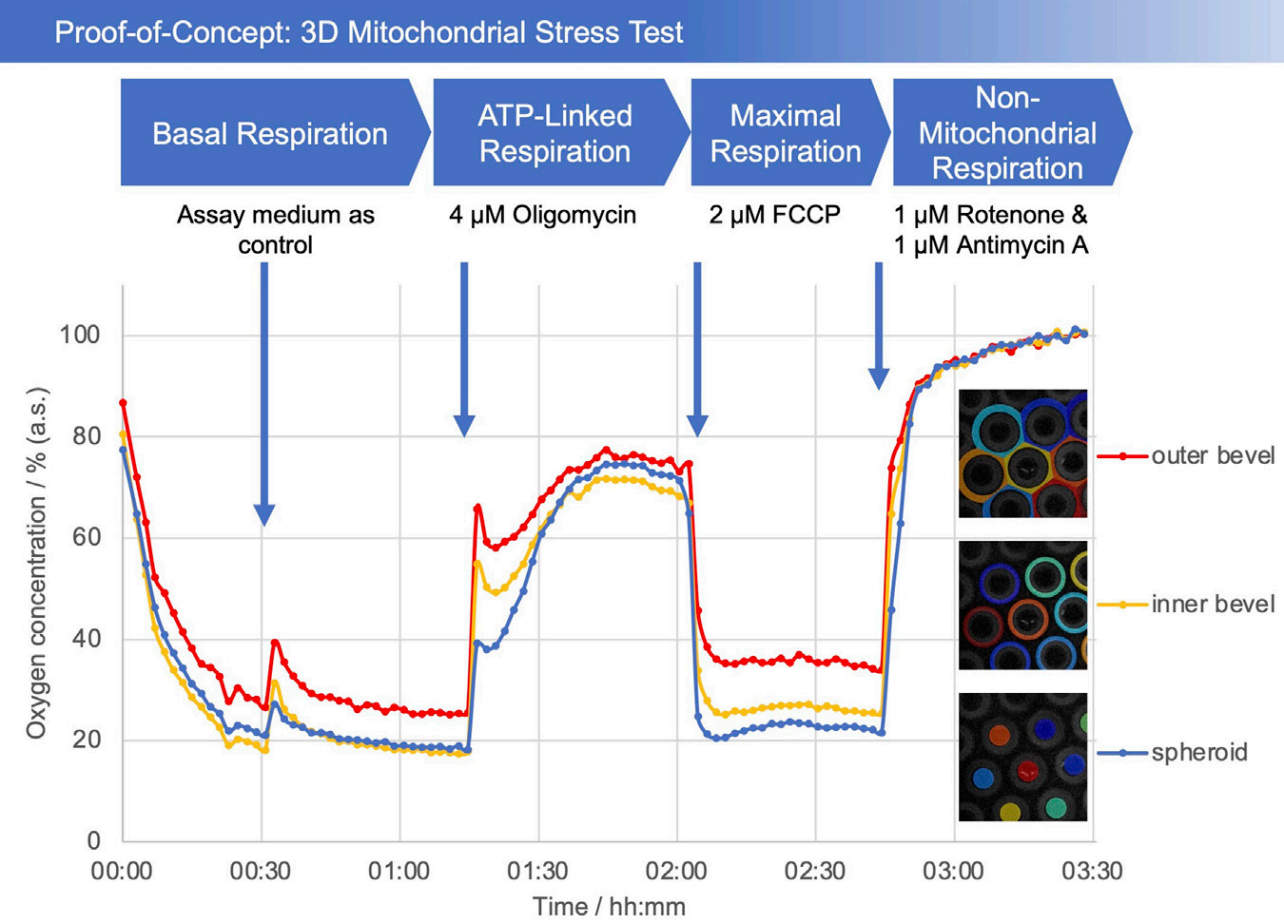
Plot of oxygen concentration in the sensor array (average of the number of the detected microcavities) during the mitochondrial stress test. 4 μM oligomycin, 2 µM FCCP, and 1 µM of rotenone and antimycin A, respectively, were added. The oxygen values are measured with open cavities, no closed microcompartment is formed and oxygen is not consumed during the measurement process. The respective spheroid in its cavity generates an oxygen (post) diffusion equilibrium with the medium, thus is in a stress-free state under optimal cultivation conditions. Closed microcompartments, on the other hand, induce temporary hypoxic (stress) situations by cutting off oxygen post-diffusion (e.g., OCR measurements Seahorse/Agilent), even the measurement process itself can result in hypoxia when polarographic sensors (electrodes) are used. Methodically or sensorically induced hypoxia is avoided with the new method, furthermore hyperoxic situations can be detected by online monitoring and controlled, e.g., *via* incubator gassing changes. The overall outcome is that the spheroid can be cultured stress-free and physioxically, so that the mitochondrial stress caused by the test compounds can be optimally quantified.

As a proof-of-concept, a so-called mitochondrial stress test was performed. The addition of medium after a 30 min incubation period served as a control. For the assay, a test substance was applied to investigate its effect on mitochondrial respiration. In the initial phase of the test, a decrease in the oxygen concentration can be seen (Figure 4). The addition of the medium causes the oxygen concentration to increase briefly, as expected. Afterwards, it levels off at around 25% in the bevel area and 20% in the spheroid area (values are relative to the maximum soluble oxygen concentration). After the addition of oligomycin, the oxygen concentration rises to over 70%, reflecting the reduced oxygen consumption of the cells. FCCP, as an uncoupling agent, leads to a decrease in the oxygen concentration (Bertholet et al., 2022; Campioni et al., 2022). After addition of rotenone and antimycin A, all mitochondrial respiration is blocked, causing the dissolved oxygen concentration to reach a maximum. Most interestingly, the difference in oxygen concentrations between the bevel and the spheroid can clearly be differentiated.

## 4 Discussion

The introduced system allows the determination of oxygen in the direct microenvironment of cells in 3D aggregates/organoids. Therefore, for the first time, a mitochondrial stress test in 3D is possible with this system. Until now, this was limited to monolayers, i.e., 2D cell cultures. Spheroids could also be studied with these standard methods. However, here, too, measurements could only be made above the cells at a single point (Campioni et al., 2022). By measuring up to 16 data points (spheroids) per measurement time point, significantly more data is generated than with measurement systems that only measure oxygen globally in the entire cultivation unit. This allows a more detailed statistical analysis.

In the proof of concept experiments, we could show that the oxygen concentrations increase or decrease as expected after addition of the respective test substances. Since the last substance cocktail (rotenone/antimycin A) inhibits complexes I and III and thus blocks the complete respiratory chain, only non-mitochondrial respiration remains, which is, however, negligible compared to mitochondrial respiration (Buttgereit et al., 2022). This allowed us to normalize our curves to an average of the last 10 measurement data points to compensate for any deviations due to differences in exposure. This was performed under the assumption that with inhibited cellular respiration, oxygen diffuses into the medium, resulting in a maximally saturated state. As previously noted, a large difference in oxygen concentrations can be observed between the region of the bevel and that of the spheroid, especially after the addition of FCCP. This can be explained by the establishment of an oxygen gradient, since more oxygen is consumed in the area of the spheroid than can be delivered by diffusion, causing the oxygen concentration to drop.

In this study, we showed the principal suitability of oxygen measurements in 3D with HepG2 spheroids. Of course, other cell types for the generation of aggregates/organoids can be used as well. By combining plasma-treated films with a collagen coating, it is possible to culture adherent cells directly on the polymer surface (Figure 2C). The characterization of spheroids produced by other methods is also possible, shown here by means of the spheroids generated by hanging drops. Due to the flexibility of the thermoforming process, other geometries of the microcavities are possible, e.g., to cultivate larger spheroids up to organoids in the sensor arrays. The size limitation then depends on the nutrient supply of the spheroids and not on the measurement method since we measure the dissolved oxygen concentration in the direct microenvironment of the spheroids/organoids and not within the aggregate. The use of a cell repellent coating, in this case BIOFLOAT™, allows the generation of spheroids directly within the microcavities. This shortens the workflow, minimizes error potentials, and allows experiments to be carried out more reproducibly.

As explained at the beginning, the tissue-relevant oxygen concentration (physioxia) plays a crucial role in cell culture. Cell culture experiments are often performed under ambient atmosphere, which corresponds to an oxygen concentration of 18.6% in the standard incubator with 5% CO_2_ (Wenger et al., 2015; Alva et al., 2022b). However, this is in no way comparable to physiological conditions and can lead to hyperoxia. On the other hand, especially in 3D cell cultures, too low an oxygen concentration can lead to hypoxia (Alva et al., 2022a). It is therefore necessary to precisely analyze and adjust the oxygen concentration in the microenvironment of the cells as needed in order to extract tissue-typical data as, e.g., by verifying the tissue marker expression in comparison to the corresponding primary tissue. The method is also applicable for very low oxygen concentrations, so that experiments under hypoxic conditions are not only feasible but are rather the assays of choice for this system. With the sensor foil SF-RPC3 used here, oxygen concentrations of 0%–150% (air saturation, a.s.) could be measured, with a higher resolution in the direction of 0% (a.s.). The standard application range is between 0% and 100% (a.s.) with a detection limit of 0.03% (a.s.) and a precision of ±0.02% (a.s.) at 0% (a.s.) and ±0.1% (a.s.) at 100% (a.s.). In the experiments presented here, the measurement of oxygen in the microenvironment of spheroids was demonstrated for the first time. Several spheroids can be characterized in different regions of interest in parallel in one experiment, which dramatically increases the data depth and thus differs from current 2D measurement systems. This approach will be used not only to show the effect of known substances on cells, e.g., by mitochondrial stress tests, as described here, but also to test the potentially harmful characteristics of new chemical entities on mitochondrial metabolism. The measurement principle is also not influenced by cells secreting extracellular matrix material since this reflects even more normal *in vivo* tissue, does not alter the oxygen signal, and therefore, increases the comparability and transferability of the results to the *in vivo* situation. Finally, by being able to continuously measure the oxygen concentration in the system, we think of feedback-regulated systems that adjust the oxygen level according to tissue needs, i.e., marker expression resembles that of primary tissue. This would be a decisive step towards the goal of establishing physioxia in cell culture.

## Data Availability

The raw data supporting the conclusion of this article will be made available by the authors, without undue reservation.
